# Measles Outbreak in Children: Experience of the Children's Hospital of Rabat (2024-2025)

**DOI:** 10.7759/cureus.102886

**Published:** 2026-02-03

**Authors:** Khadija Belcadi Abassi, Aminou Sara, Khadija Ilyla, Soumaya Benchekroun, Zouheir Meiouet, Naima El Hafidi, Chafik Mahraoui

**Affiliations:** 1 Department of Pediatric Pneumo-Allergology and Infectious Diseases, Children’s Hospital of Rabat, Mohammed V University, Rabat, MAR; 2 Department of Medical Affairs, Epidemiological Surveillance Unit, Rabat, MAR

**Keywords:** children, complications, generalized rash, measles, measles complication, measles in children, measles virus infection, outbreak, vaccination, vaccine

## Abstract

Introduction: Measles remains a highly contagious viral disease and a persistent public health challenge despite the availability of effective vaccination. Since late 2023, Morocco has experienced a resurgence of measles cases, highlighting gaps in vaccination coverage and surveillance. This study aimed to describe the epidemiological, clinical, and outcome characteristics of children hospitalized for measles during the 2024-2025 outbreak at the Children’s Hospital of Rabat and to compare the occurrence of complications according to vaccination status and age group.

Materials and methods: A retrospective hospital-based observational study was conducted in the Department of Pediatric Pneumo-Allergology and Infectious Diseases at the Children’s Hospital of Rabat between February 1, 2024, and September 1, 2025, corresponding to the measles outbreak period. Sociodemographic, clinical, paraclinical, and outcome data were extracted from medical records. Categorical variables were summarized as frequencies and percentages. Continuous variables were summarized using medians and interquartile ranges, given the skewed age distribution. Comparisons were performed using the chi-square or Fisher’s exact test.

Results: A total of 587 children were included, with a median age of 15 months; 67% were infants. Boys accounted for 57% of cases, and 56% of children lived in rural areas. Overall, 61% were unvaccinated, 16% were partially vaccinated, and 23% were fully vaccinated. Complications occurred in 56% of patients, predominantly respiratory (54%). Complications were more frequently observed among children who were not fully vaccinated compared to fully vaccinated children.

Conclusion: This study provides a descriptive hospital-based overview of pediatric measles cases requiring admission during the 2024-2025 outbreak. Severe complications and in-hospital mortality were frequently observed among hospitalized children, particularly among younger infants. These findings are limited to hospitalized cases and do not allow inference regarding vaccine effectiveness, outbreak dynamics, or population-level disease severity.

## Introduction

Measles is an acute and highly contagious viral infection caused by a Morbillivirus, with humans as its only reservoir [1]. Despite the widespread availability of effective vaccines and the substantial reduction in morbidity and mortality following vaccine introduction in 1963, measles remains a major public health concern, particularly in low- and middle-income countries. In Africa, several countries, including Liberia, Madagascar, and the Democratic Republic of Congo, continue to experience recurrent epidemic outbreaks [2].

In 2018, the World Health Organization (WHO) estimated nearly 9.8 million measles cases and 140,000 related deaths worldwide, most of which occurred in children under five years of age [3]. Fragile health systems, population displacement, and interruptions in routine immunization programs-further aggravated by the COVID-19 pandemic-have contributed to the re-emergence of measles in multiple regions [4].

In Morocco, measles vaccination was introduced in 1982 as part of the Expanded Program on Immunization (EPI). The strategy was reinforced by the introduction of a second vaccine dose in 2014, along with nationwide mass vaccination campaigns conducted in 2008 and 2013, according to data from the Ministry of Health and Social Protection, Directorate of Epidemiology and Disease Control (unpublished internal report, Rabat, 2024). Despite these efforts, a resurgence of measles cases has been observed since September 2023, highlighting persistent gaps in vaccination coverage and the need for strengthened epidemiological surveillance and timely clinical management to prevent severe complications such as pneumonia and encephalitis.

Study objectives

The primary objective of this study was to describe the epidemiological, clinical, and outcome characteristics of children hospitalized for measles during the 2024-2025 outbreak at the Children’s Hospital of Rabat.

The secondary objectives were to descriptively compare the occurrence of complications, intensive care unit admission, and in-hospital mortality according to vaccination status and age group.

## Materials and methods

Study setting

This study was conducted in the Department of Pediatric Pneumo-Allergology and Infectious Diseases at the Children’s Hospital of Rabat (HER), a national tertiary referral center specializing in the management of complicated pediatric infectious diseases. The hospital receives patients from the Rabat-Salé-Témara region as well as referrals from other regions of Morocco, encompassing both urban and rural populations. The department is equipped with dedicated pediatric inpatient wards and has access to on-site laboratory facilities and pediatric radiology services, which are routinely used for the diagnosis, monitoring, and management of infectious diseases, including measles.

Study design

This was a retrospective, single-center, hospital-based observational case series conducted over a 19-month period from February 1, 2024, to September 1, 2025, corresponding to the national measles outbreak. The study population included all children aged between two months and 15 years who were hospitalized for measles during the study period. An exhaustive sampling strategy was applied, whereby all consecutive medical records meeting the eligibility criteria were included to minimize selection bias and ensure representativeness of hospitalized cases.

Inclusion criteria

Children aged two months to 15 years who required hospitalization for measles during the study period were included. Eligible patients met the definition of a suspected case, laboratory-confirmed case, epidemiologically linked case, or clinically compatible case of measles, in accordance with the Operational Plan for Measles Surveillance and Outbreak Response (2024).

Exclusion criteria

Patients managed exclusively on an outpatient basis without hospitalization were excluded. Patients who left the hospital against medical advice before completion of clinical assessment and inpatient management were also excluded due to incomplete clinical, paraclinical, or outcome data.

Case definitions and classification

Classification into laboratory-confirmed, epidemiologically linked, or clinically compatible cases was performed according to the national Operational Plan for Measles Surveillance and Outbreak Response (2024). Laboratory-confirmed cases required a positive measles IgM (immunoglobulin M) result or RT-PCR (reverse transcription polymerase chain reaction). Epidemiologically linked cases were defined as suspected cases with documented contact with a laboratory-confirmed case within the recognized transmission period. Clinically compatible cases were those fulfilling the clinical case definition in the absence of laboratory confirmation or documented epidemiological linkage.

Measles IgM testing was performed as part of routine clinical care at the hospital laboratory, in accordance with national diagnostic recommendations in effect during the outbreak period. As this study was retrospective, detailed information regarding assay manufacturer, sensitivity, specificity, or exact timing of sampling relative to rash onset was not consistently available and therefore could not be analyzed.

Data sources and data collection procedures

Data were retrospectively extracted from inpatient medical records, including admission notes, daily clinical follow-up sheets, nursing charts, laboratory results, radiology reports, and discharge summaries. Data extraction was performed using a standardized data collection form to ensure consistency in variable definitions and recording. Each patient was assigned a unique anonymized study code to ensure confidentiality. Data were entered into Microsoft Excel 2019 (Microsoft Corporation, Redmond, WA, USA) using predefined coding rules, followed by systematic verification to identify and correct potential entry errors.

Variables and operational definitions

Collected sociodemographic variables included age (in months or years), age group (infants, 2-6 years, and >6 years), sex, area of residence (urban or rural), and geographic origin (region or province when available). Vaccination status was classified into three categories based on documentation in medical records and/or vaccination cards when available: unvaccinated (no dose received), partially vaccinated (one dose received or incomplete schedule for age), and fully vaccinated (schedule complete for age, including two doses when applicable). Vaccination status was determined preferentially from vaccination cards. In the absence of documented records, parental recall was used. When discrepancies existed, documented vaccination records were considered authoritative. The proportion of cases classified based on parental recall could not be reliably quantified retrospectively.

Clinical presentation variables included symptoms and signs at admission, such as fever, cough, rhinorrhea (coryza), conjunctivitis, enanthem, Koplik spots, and rash characteristics (maculopapular nature, generalized or localized distribution, cephalocaudal progression, and palmoplantar involvement). When available, time intervals were recorded, including delays between fever onset and rash appearance, and between symptom onset and first medical consultation.

Paraclinical data included biological and radiological findings. Biological parameters comprised C-reactive protein (CRP) categorized as ≤10 mg/L, 10-50 mg/L, or >50 mg/L, as well as the presence of lymphopenia, hyponatremia, anemia, thrombocytopenia, and hepatic cytolysis when documented. Radiological data were based on chest X-ray findings, classified as normal or abnormal; abnormal findings were further categorized into interstitial, alveolar, bronchoalveolar, or other patterns as reported by the radiologist.

Respiratory complications were defined based on clinical diagnosis documented by the treating physician and supported, when available, by radiological findings. Pneumonia was defined as the presence of respiratory symptoms associated with radiological abnormalities consistent with infection. Neurological complications referred to clinically diagnosed encephalitis documented in medical records. Severe complications were defined as the occurrence of at least one respiratory, neurological, or digestive complication requiring inpatient management.

Clinical management, including antibiotic prescription, admission to the intensive care unit, and supportive treatments such as oxygen therapy or rehydration, followed routine local clinical practice and physician judgment. No standardized management protocol was applied during the study period, and treatment decisions reflect real-world care during the outbreak.

Management of missing data and data quality

Variables not documented in the medical records were treated as missing and were not imputed. For each analysis, denominators corresponded to the number of patients with available data for the variable of interest. To enhance data quality, the dataset was reviewed after data entry to identify inconsistencies, such as incompatible age categories, duplicated records, or implausible values, which were corrected by rechecking the source medical records. Analyses were performed using available-case analysis. Variable-specific denominators were used when data were missing, and no imputation was performed.

Statistical analysis

Statistical analyses were descriptive and comparative in nature. No multivariable modeling or adjustment for confounders was planned or performed. Comparative analyses were conducted to explore associations between vaccination status or age group and selected outcomes. Statistical analysis was conducted using JAMOVI software (version 2.5, 2024). Categorical variables were summarized as frequencies and percentages. Continuous variables were summarized using medians and interquartile ranges, given the skewed age distribution. Comparisons were performed to explore associations between vaccination status and the occurrence of complications, as well as between age group and the occurrence of complications. The Chi-square test was used to compare proportions, or Fisher’s exact test when expected cell counts were small. Test statistics (χ² values) and p-values were reported. Statistical significance was defined as p<0.05. No prospective study protocol or preregistration was performed, as the study was conducted retrospectively using routinely collected clinical data.

Ethical considerations

The study protocol was approved by the Institutional Review Board of the Faculty of Medicine and Pharmacy of Rabat. Given the retrospective design using anonymized routine-care data, the requirement for informed consent was waived. Confidentiality was ensured through anonymization of extracted data and restricted access to the study database.

## Results

The study included 587 hospitalized children with measles, with a median age of 15 months. Infants accounted for 67% of cases. A male predominance was observed, with 57% boys and 43% girls. The majority of cases originated from rural areas (56%). The most affected regions were Salé (24%), followed by Rabat and Témara (21%), and Skhirat (15%). Diagnosis was established through an epidemiological link in 57% of cases, laboratory confirmation in 21%, and clinical compatibility criteria in 22%.

Table 1 presents the distribution of cases according to age, sex, area of residence, and method of confirmation.

**Table 1:** Distribution of confirmed measles cases according to age group, sex, area of residence, and type of confirmation

Variables	Number	Percentage
Age		
Infants	393	67%
Between 2 and 6 years	129	22%
>6 years	65	11%
Sex		
Boys	334	57%
Girls	250	43%
Residence area		
Rural	329	56%
Urbain	258	44%
Type of confirmation		
Epidemiological link confirmation	335	57%
Laboratory confirmation	123	21%
Clinically compatible case	129	22%

**Figure 1:**
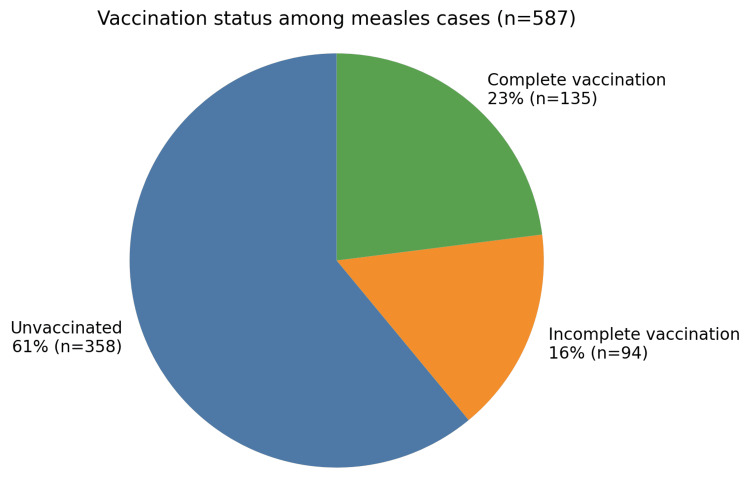
Distribution of confirmed measles cases according to vaccination status

Regarding vaccination status, 61% of children were unvaccinated, 16% had an incomplete vaccination status, and 23% were fully vaccinated (Figure 1). 

**Figure 2:**
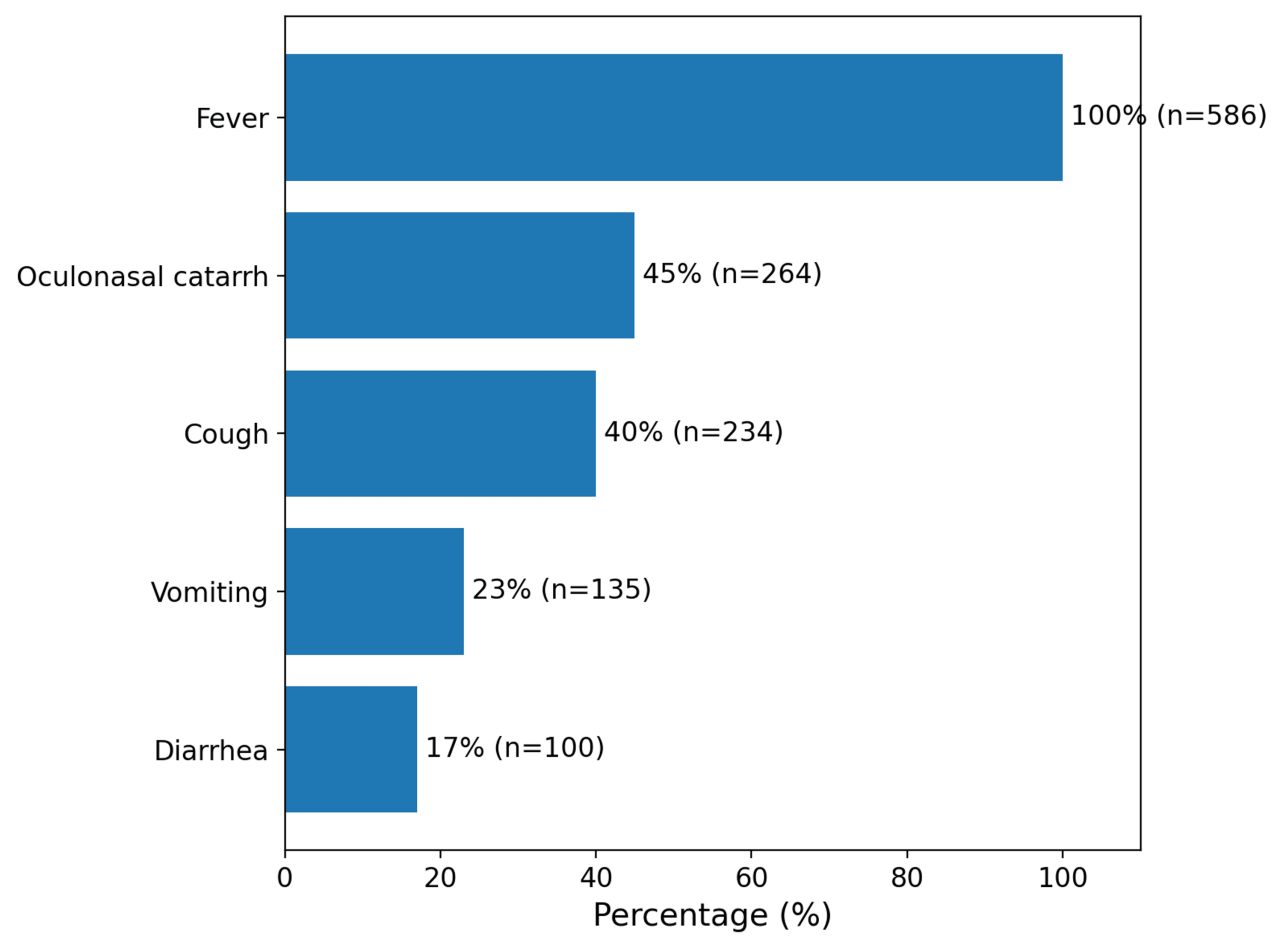
Initial symptomatology of confirmed measles cases

The main initial symptoms observed are presented in Figure 2. Fever was constant in all patients, followed by oculonasal catarrh (45%) and cough (40%). Medical consultation occurred on average five days after the onset of the first symptoms, while the skin rash appeared approximately 3.8 days after the onset of fever.

Clinically, fever and rash were present in all patients. Conjunctivitis was observed in 91%, cough in 73%, enanthem in 48%, rhinorrhea (coryza) in 36%, and Koplik’s spots in 17% of cases (Table 2). The rash had a cephalocaudal progression in all patients, maculopapular in 91% of cases, generalized in 81%, and associated with palmoplantar involvement in 50%.

**Table 2:** Clinical manifestations of patients with measles

Clinical symptoms	Percentage	Number
Exanthema	100%	587
Enanthema	48%	282
Koplik’s spots	17%	100
Conjunctivitis	91%	534
Coryza	36%	211
Cough	73%	428

**Figure 3:**
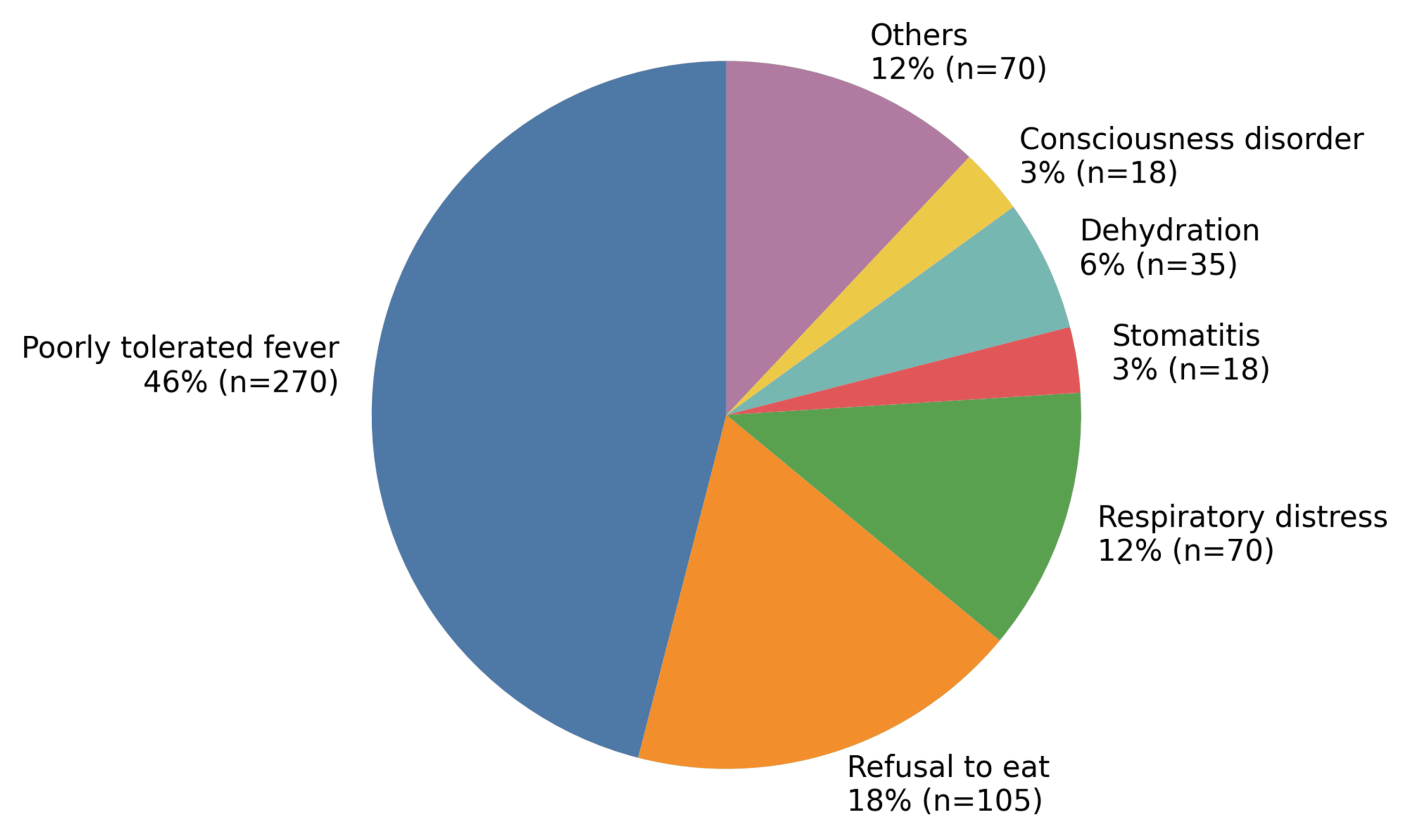
Reasons for hospitalization among children with measles

The main reasons for hospitalization are shown in Figure 3. Fever that was poorly tolerated was the most frequent reason for hospitalization (46%), followed by refusal to eat (18%). Other causes, such as dehydration, stomatitis, and respiratory distress, were less frequent.

Regarding radiological findings, 75% of patients had normal chest X-rays, while interstitial syndromes (6%), alveolar syndromes (4%), and bronchoalveolar syndromes (1.6%) were observed in the remaining cases.

Biologically, the most common abnormalities were lymphopenia (20%), hyponatremia (17%), and hypochromic microcytic anemia (14%). Other abnormalities, such as thrombocytopenia and hepatic cytolysis, were observed at lower rates.

CRP assessment showed that 64% of patients had CRP ≤10 mg/L, 28% had CRP between 10 and 50 mg/L, and 8% had CRP >50 mg/L. No statistically significant association was found between CRP levels and vaccination status.

**Figure 4:**
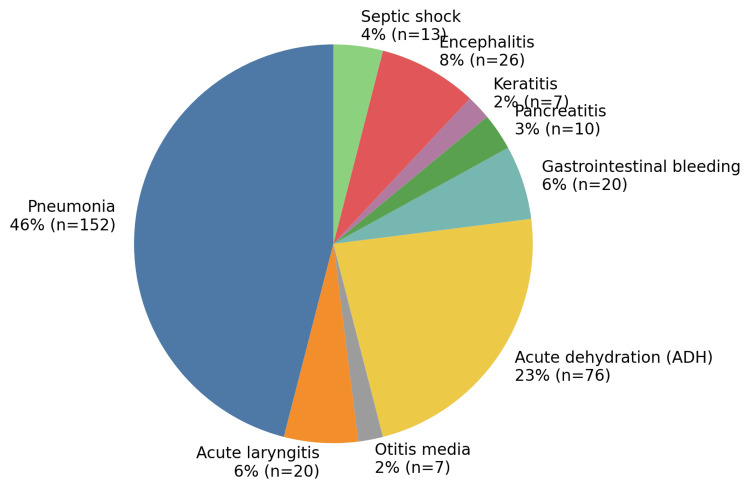
Distribution of complications associated with measles among hospitalized patients

Severe complications affected 330 patients (56%), mainly dominated by respiratory forms (54%), followed by digestive complications (32%). Specific respiratory complications included pneumonia (46%), acute laryngitis (6%), and acute otitis media (2%). Digestive complications were primarily represented by acute dehydration (23%) and gastrointestinal bleeding (6%). Neurological complications were mainly encephalitis (8%). All these data are illustrated in Figure 4.

The analysis of complications according to vaccination status showed that children who were not fully vaccinated were significantly more likely to develop complications (63%) compared to fully vaccinated children (33%), with a statistically significant difference (p<0.001). Among the partially or unvaccinated patients, 286 developed complications, compared to only 44 among fully vaccinated children (Table 3).

**Table 3:** Correlation between vaccination status and the occurrence of complications in patients with measles Data are presented as n (%). Comparison between groups was performed using the Chi-square test (χ²=38.88, df=1, p<0.001).

Vaccination status	No complications, n (%)	Complications, n (%)	P-value
				Not fully vaccinated	165 (36.6%)	286 (63.4%)	-
-						
Fully vaccinated	91 (67.4%)	44 (32.6%)	-
			-
Total	256 (43.7%)	330 (56.3%)	<0.001

Similarly, the frequency of complications decreased significantly with age (p=0.026). The risk was highest among infants and gradually decreased in older children (Table 4). 

**Table 4:** Correlation between age and the occurrence of complications in patients with measles Data are presented as n (%). Comparison between groups was performed using the Chi-square test (χ²=16.52, df=2, p<0.001).

Age group	No complications, n (%)	Complications, n (%)	P-value
Infants	120 (36.4%)	210 (63.6%)	-
2-6 years	70 (53.8%)	60 (46.2%)	-
>6 years	66 (52.4%)	60 (47.6%)	-
Total	256 (43.7%)	330 (56.3%)	0.02

Treatment was primarily symptomatic. Vitamin A was administered in 78% of cases within the first two days following admission. Antipyretics, oral or intravenous rehydration, and oxygen therapy were prescribed as clinically indicated. Additionally, 20% of patients received antibiotic therapy due to suspected or confirmed bacterial co-infections.

The median duration of hospitalization was six days. Most children (94%) were managed in the pediatric ward, while 6% required transfer to the intensive care unit. The overall case-fatality rate was 3%, involving exclusively unvaccinated children, with a median age of nine months.

## Discussion

This study provides a descriptive overview of pediatric measles cases requiring hospitalization during the 2024-2025 outbreak at a tertiary referral center in Rabat. The findings reflect the clinical profile, complications, and in-hospital outcomes observed among admitted children and should be interpreted strictly within the context of a hospital-based cohort.

By design, this study systematically excludes mild and outpatient measles cases and therefore overrepresents severe clinical presentations, limiting the generalizability of complication rates, intensive care use, and mortality beyond hospitalized children.

Measles is an extremely contagious viral disease caused by an RNA virus from the Paramyxoviridae family. It is transmitted through airborne droplets or direct contact with infected secretions, primarily affecting children and potentially leading to serious or even fatal complications. With a strictly human reservoir, the measles virus has a very high basic reproduction number, estimated between 18 and 20, making it one of the most transmissible infectious diseases [5,6]. The incubation period generally ranges from 7 to 14 days. Contagiousness begins two to five days before the appearance of the rash and continues for five to six days after [7]. Immunity against measles can be acquired naturally after infection or through vaccination. IgM antibodies appear at the onset of the rash and persist for about a month, while IgG antibodies, detectable a few days later, provide long-term protection [8]. The initial symptoms include runny nose, cough, high fever, and conjunctivitis. These signs precede the characteristic rash, which starts on the face before spreading to the rest of the body, lasting an average of five to six days [7]. Common complications such as otitis and diarrhea are frequently reported, whereas more severe forms - pneumonia, encephalitis, and subacute sclerosing panencephalitis (SSPE) - though rare, can be fatal [9]. In Morocco, measles vaccination was incorporated into the Expanded Program on Immunization (EPI) in 1982, with a single dose administered at 9 months of age. Although national vaccination coverage has exceeded 90% since 1995, cyclical epidemics continue to occur. This persistence is mainly due to the partial efficacy of the vaccine at that age (estimated at 85%) and the progressive accumulation of susceptible children [5]. To address this limitation, a second vaccine dose was introduced in 2003 for children aged 6 years and later moved to 18 months in 2014. Additionally, mass vaccination campaigns were conducted in 2008 and 2013 [5]. These combined efforts led to a substantial reduction in measles morbidity and mortality.However, the COVID-19 pandemic negatively impacted vaccination coverage and epidemiological surveillance, leading to decreased immunization rates and underreporting of febrile rash cases, further exacerbating existing vulnerabilities [10].

Our findings confirm current epidemiological concerns and highlight local specificities while remaining consistent with international trends. Among the studied cases, 67% were infants, a proportion higher than that reported in Mali (48%) [11] and Italy (50%) [12]. This increased vulnerability among infants may be explained by the rapid loss of maternal antibodies, particularly after weaning. A male predominance (57%) was also observed, consistent with data from Mali (53%) [11] and Greece (54%) [13]. However, higher proportions were reported in India (70%) [14] and Mauritania (60%) [15], possibly due to biological, sociocultural factors, or inequalities in access to healthcare. Vaccination status plays a decisive role in measles prevention. In our study, 77% of hospitalized children were not fully vaccinated, a figure similar to those reported in India (84%) [14] and Mauritania (89%) [15].

Observed differences according to vaccination status represent unadjusted descriptive associations within a hospitalized cohort and should not be interpreted as evidence of vaccine effectiveness or protection against severe disease, as these comparisons are highly confounded by age, baseline severity, comorbidities, and healthcare-seeking delay.

Respiratory complications were the most frequent and severe clinical manifestations in our cohort, affecting 54% of hospitalized patients, mainly in the form of pneumonia. These observations are consistent with findings from Mauritania (53%) [15] and India (82%) [14]. Pneumonia, whether due to bacterial superinfection or direct viral injury, remains the leading cause of measles-related mortality, particularly among malnourished children. Vitamin A deficiency and immunosuppression further aggravate this risk. Our findings support the World Health Organization (WHO) recommendations, which advocate for the systematic administration of vitamin A to reduce measles-related morbidity and mortality [16].

The most frequently observed paraclinical abnormalities in our study included elevated CRP (36%), lymphopenia (20%), hyponatremia (17%), and thrombocytopenia (11%). These findings reflect an intense inflammatory response and a weakened immune status. Our results are consistent with those reported in other contexts, notably in Mali [11] and Greece [13,17].

The broader public health context of measles resurgence is provided for background purposes only and is not derived from the present hospital-based data.

Limitations of the study

This study has several important limitations. First, its hospital-based design introduces a major selection bias, as only children requiring hospitalization were included. Consequently, the findings cannot be generalized to all measles cases in the community, and observed rates of complications, intensive care admission, and mortality likely overestimate the true burden of severe disease.

Second, case confirmation was heterogeneous, with a substantial proportion of cases classified as epidemiologically linked or clinically compatible without laboratory confirmation, introducing diagnostic uncertainty and potential misclassification.

Because fewer than one quarter of cases were laboratory confirmed, diagnostic misclassification cannot be excluded, which may have influenced reported symptom profiles, complication rates, and outcome distributions.

Third, comparisons according to vaccination status were unadjusted and subject to significant confounding by age, baseline severity, comorbidities, and healthcare-seeking delays. These analyses do not support causal inference regarding vaccine effectiveness.

By design, this study systematically excludes mild and outpatient measles cases and therefore overrepresents severe clinical presentations, limiting the generalizability of complication rates, intensive care use, and mortality beyond hospitalized children.

Finally, the absence of population denominators, incidence data, or temporal trend analysis precludes any conclusions regarding outbreak dynamics or epidemic control.

## Conclusions

This retrospective, single-center, hospital-based study provides a descriptive overview of pediatric measles cases requiring hospitalization during the 2024-2025 outbreak. The findings characterize the clinical presentation, complications, intensive care utilization, and in-hospital mortality observed among admitted children, with severe outcomes predominantly affecting younger infants.

These results are restricted to hospitalized cases and should not be generalized to all pediatric measles infections. The study does not allow inference regarding vaccine effectiveness, outbreak dynamics, population-level disease severity, or epidemic control. Its contribution lies in documenting real-world clinical patterns of severe pediatric measles encountered in hospital practice during an outbreak.
